# QM/MM Dynamics Study of the Augmenting Effects of Reduced Graphene Oxide Towards the Butadiene Acrylonitrile Copolymer Matrix and Self-Repair of the Enhancer

**DOI:** 10.3390/nano16020113

**Published:** 2026-01-15

**Authors:** Dobromir A. Kalchevski, Stefan K. Kolev, Kamen V. Ivanov, Dimitar A. Dimov, Aneliya S. Kostadinova, Hristiyan A. Aleksandrov, Teodor I. Milenov

**Affiliations:** 1 ”E. Djakov” Institute of Electronics, Bulgarian Academy of Sciences, 72 Tzarigradsko Chausee Blvd., 1784 Sofia, Bulgariaivanov@snakesoft.eu (K.V.I.); dadimov@ie.bas.bg (D.A.D.);; 2 Institute of Biophysics and Biomedical Engineering, Bulgarian Academy of Sciences, 1113 Sofia, Bulgaria; 3 Faculty of Chemistry and Pharmacy, Sofia University “St. Kliment Ohridski”, 1 J. Bourchier Blvd., 1164 Sofia, Bulgaria; haa@chem.uni-sofia.bg

**Keywords:** QM/MM, rGO nanoparticle, butadiene–acrylonitrile copolymer, self-repair, dynamics

## Abstract

This study utilizes QM/MM Born–Oppenheimer Molecular Dynamics in order to model the process of intermolecular binding between reduced graphene oxide (rGO) and butadiene–acrylonitrile copolymer (PBDAN) with a monomer ratio of 2:1. This research aims to elucidate the structural reasons behind the enhancing properties of the substrate, focusing on the polymer matrix. The behavior of each phase was examined and discussed. More importantly, the intermolecular interactions within the interphase zone of adsorption were investigated on an atomic scale. We found and characterized 58 such instances, grouped into hydrogen bonds and three types of stacking: π–π, σ–π, and σ–n. Each occurrence was analyzed through the use of radial distribution functions. Five spontaneous chemical processes within the rGO nanoparticle were modeled and characterized. Two of them were found to provide stabilization only within the substrate, while the rest are relevant for the overall constitution of the heteromaterial. Perhaps most intriguing is the process of self-repair as part of the vacancy defect. This occurs entirely within the carbon frame of the rGO layer. We believe our results to be of importance for a large set of ligand materials, mostly those which contain unsaturated bonds and electronegative atoms.

## 1. Introduction

Augmenting organic polymers with graphene-based materials is a novel research area in materials science. The introduction of small amounts of reduced graphene oxide (rGO) can controllably adjust a wide variety of key physical and physicochemical properties. The resulting nanocomposites may even gain unique characteristics. The usual targets of augmentation vary from tensile strength [1,2], storage modulus [1,2], work function [3] through thermal stability [4,5], and thermal conductivity [6,7] to electric conductivity [8,9], electromagnetic shielding [10,11], and optoelectronic properties [12,13]. Altered degrees of compaction [14] and hydrophobicity [15] have also been observed. Lab-ready rGO/polymer coatings have potential contributions in various fields, as they exhibit the following properties: water repellence [14], water desalination [16], biocompatibility [17], resistance to corrosion [18], antibacterial activity [19], and self-repair [20,21]. Common potential applications of rGO/polymer heterostructures include the following: mechanical and chemical stability [22], selective molecular capture [23], molecular sensors [24], 3D printing [13], osteogenesis [25], gas barriers [26], membrane separation [27], nanofluidic technology [28], supercapacitors [29], and energy storage [25,30,31].

The present study is motivated by parallel experimental research in which we synthesized carbon composites based on PBDAN and SPHERON 5000 carbon black (CB), treated with acetone. Previous research, conducted by ourselves [32], shows that rGO is the closest structural and morphological analogue to acetone-functionalized CB. Details of the preparation can be found in [32]. The aim of this experimental work was to develop an elastomer whose electrical resistivity changes in an almost linear fashion within the desired pressure range (4–15 kPa). One possible application is the manufacturing of high-precision pressure sensors. The carbon black content varied between 0 and 120 parts per hundred resin (PHR). An optimal concentration of 70 PHR was identified for the intended application. The research is currently in its final stages, and will be published soon. Preliminary results have been presented at the Twenty-Third International Conference and School on Quantum Electronics: Laser Physics and Applications [33].

Studies regarding the preparation and property assessment of such nanocomposites rarely involve thorough (if any) understanding of the behavior of the given material, in terms of atomic structure. Theoretical investigations into the problem are rather scarce and often seek non-fundamental understanding. Stancovic et al. investigated the effects of functionalization of a GO/polymer composite, regarding CO_2_ capture [23]. According to their study, the presence of GO was found to be irrelevant for gas adsorption. The authors’ explanation was that a remarkably powerful GO/copolymer interaction limits the stabilization of CO_2_/copolymer binding. Keshmiri et al. found that the presence of Zn-aminotrimethylene phosphonic acid-rGO nanoparticles improves the thermal and mechanical characteristics of an epoxy matrix [2]. Kritikos et al. studied the behavior of poly(acrylic acid) and polystyrene chains bound to graphene-based nanosheets [34]. The authors concluded that there is a higher degree of anisotropy in polymer diffusion, when the substrate is pristine graphene. Inter-phase binding appeared to exist under the laws of Arrhenius’ equation. The macromolecular chains beyond the adsorbed layer were found to behave in a fashion characteristic of the pure polymer. The study proposes that the improvement in tensile strength and elongation break point were due to a good interfacial binding with the rGO. A review by Srivastava et al. summarizes the increase in elastic modulus, modulus of rigidity, and hardness of nitrile butadiene rubber due to the introduction of graphene oxide [35]. Yang et al. utilized MD to predict the pull-out and peel-off stress required to separate GO and modified GO (treated with 3-aminopropyl-triethoxysilane) from nitrile butadiene rubber [36]. It was estimated that GO was easier to pull, yet it was harder to peel. The difference in properties between the two graphene derivatives was attributed to an uneven amount of hydrogen bonds. Song et al. investigated the compatibility and damping performance of a GO-grafted antioxidant/nitrile-butadiene rubber composite through classical molecular dynamics (MD) [37]. According to their model, the addition of GO increased damping performance and binding energy, while reducing the free volume fraction of the composite. Li et al. investigated the mechanical and tribological properties of oxide-reinforced polyamide 66/nitrile-butadiene rubber composites augmented with graphene [38]. The high surface area of graphene and the associated large vdW forces were stated as pivotal factors in the improvement of its mechanical properties. There was a noticeable reduction in cohesive energy density, mean square displacement, free volume, friction coefficient, and wear rate.

According to our literature review, previous publications regarding the topic of heteromaterials containing graphene and graphene-like phases ignore most non-covalent types of interaction. Although π–π stacking has been examined [39,40,41], no characterization has been performed in terms of σ–π, σ–n, or n–π interactions. Such forms of stabilization are of great importance in the field of macromolecular chemistry. Additionally, all previous research is based on either geometrical optimization or classical molecular dynamics. Exploring polymer creasing and entanglement through static calculations is a nearly impossible task due to the lack of systematic predictability in macromolecular motion. Furthermore, spontaneous chemical processes cannot be modeled outside of the framework of time. Transition state optimization can only be conducted over a strictly predetermined mechanism (regardless of whether it is the most likely or at least plausible). Such an approach always includes the chance that a probable process will be omitted. It would require multiple separate research studies for any attempt to include all possible reactions within a multilayered rGO system of a realistic size. Besides the multitude (and kinds) of substituents, the lack of reasonable prediction in interlayer shifts prohibits knowing which functional groups would participate in chemical processes between layers. As for classical mechanics, is the fact remains that simulating a molecular system without a wavefunction equals an attempt to represent it outside of its real/natural form. Such oversimplification only allows for the prediction of a limited set of properties. The effects of intermolecular stacking can only be modeled within a paradigm involving electronic density. Most force fields cannot model chemical reactions. Additionally, the classical approach yields larger deviations compared to ab initio methods. Significant insufficiencies in the model of stabilizing interactions, whether they are covalent or not, would result in a poor prediction of all characteristics, arising from bond strength and intermolecular binding. This includes all physical properties concerning material deformation and thermal behavior.

It has been established that the electronic structure of graphene is beneficial to the self-healing of adsorbed phases in composite materials [42,43,44]. Theoretical modeling has been employed in order to clarify such processes [45,46]. The intrinsic self-healing of graphene phases is also well known [47,48]. A few studies have simulated the self-repair of this carbon material in terms of classical molecular dynamics with a reactive force field [49,50]. As far as we know, only one study has attempted to provide a quantum mechanical model of such processes [51], and unfortunately it does not provide a reaction sequence yielding true graphene structure or at least geometrical configuration with minimal defects. In this article, the rGO model is entirely based on quantum mechanics. We seek the geometry of maximum possible self-repair, without the availability of additional C atoms.

In the present study, we would like to find the fundamental reduced graphene oxide–polymer interactions in order to elucidate and confirm the properties specific to these heteromaterials. Investigating the electronic structure on the border region of the nanocomposites is the only way to reveal their physical and chemical properties. We employ a rare and advanced approach which (to our knowledge) has never been utilized in this field—QM/MM Dynamics. By modeling the rGO phase with an ab initio method, we can reach a more accurate description of both the folding process of the copolymer, consisting of butadiene and acrylonitrile in a ratio of 2:1, and the intermolecular binding between the two phases. Additionally, this approach allows for the occurrence and correct characterization of spontaneous reactions. Further, we employed a more experimentally realistic model of three-layer reduced graphene oxide with a rich variety of substituent-based defect augmentations. The polymer chains are approximately five times longer than any dimension of the rGO particle. The model achieves saturated adsorption on both surfaces of the substrate. The amount of copolymer is sufficient to model the gradient in polymer behavior, reaching a zone of pure PBDAN. We also characterize all present types of intermolecular interactions. We give special attention to the reactions involved in self-healing.

## 2. Methods

All calculations were performed with the CP2K/Quickstep 2025.1 package [52,53]. While the rGO phase was modeled on the Quantum Mechanical (QM) level, the polymer phase was modeled in Molecular Mechanics (MM). Electrostatic coupling was employed [54] in order to account for the regions’ mutual response during the convergence procedures. In other words, the overall electromagnetic field of nuclei and electronic orbitals is influenced by the dot charges of the polymer chains, and vice versa. In turn, the molecular structure of the QM region and the geometry of the MM chemical units are optimized in concordance. The Self-Consistent Field (SCF) iterations were completed using the Self-Consistent Charge Density Functional Based Tight Binding (SCC-DFTB/DFTB2) method [55], with a mio-0-1 parameter set [55]. An efficient a posteriori treatment for dispersion interaction was employed [56]. The classical mechanics force field employed was the latest version (as of April 2025) [57] of General AMBER Force Field 2 (GAFF2) [58]. The parametrization of the MM region was carried out with the software package Amber Tools 25. Only the default parameters were applied.

The DFTB method is an approximation to Density Functional Theory (DFT), in which the Kohn–Sham (KS) equations are transformed to tight binding ones [59] related to the Harris functional [60]. The second-order expansion of the KS equations enables a transparent, parameter-free generalized Hamiltonian matrix. Its elements are modified by a self-consistent redistribution of Mulliken charges [55]. The KS energy additionally includes the Coulomb interaction between charge fluctuations. The accuracy of the DFTB method with self-consistent charge expansion is comparable to that of DFT methods and higher levels of ab initio theory for various properties of single molecules, solutions, and solid-state materials, yielding satisfactory geometries and total energies [61,62,63]. DFTB produces realistic charge distribution and binding energies of charged solvated species [64]. The derived activation energies in organic chemistry also conform to higher levels of theory [5], enabling the study of reaction mechanisms. This method is found to yield excellent geometries and energetics for pure carbon species, such as fullerenes ranging from C_20_ to C_86_ [65]. Recently, we employed DFTB2 in a metadynamics study of the formation of fullerenes in the interstellar medium [66]. We also used this method to model the growth of sp^2^ (graphene) and sp^3^ carbon phases during high-temperature Chemical Vapor Deposition [67]. We selected SCC-DFTB in order to simulate an rGO nanoparticle with a realistic size. All DFT Tight Binding methods are at least one order of magnitude faster than PBE [61]. Although MM atoms are present in the QM explicit cell, only (and all) rGO atoms are modeled on the QM level of theory.

The simulations of the reactions are performed using Born–Oppenheimer Molecular Dynamics (BOMD) [68]. The system was computed in Periodic Boundary Conditions (PBC). In order to enable optimal compression, the time evolution of the studied system was conducted in an NPT ensemble. The thermostat utilized canonical sampling through velocity rescaling (CSVR) [69,70]. In order to model the system under experimental conditions, the temperature was set to 433 K, while the barostat was set to a pressure of 130 atm. The time step was set at 1 fs. The shortest intermolecular distances were above 3.4 Å in the initial geometry. The dynamics run was preceded by a cell and geometry optimization of the system. Temperature tolerance was always set to 50 K.

BOMD is a well-established method for the simulation of both organic and inorganic reactions in a wide range of environments and conditions [55,61,71,72,73,74].

Intermolecular interactions were visualized through 2D IRI analysis [75], as implemented in MultiWfn 3.8 [76,77]. Such figures show the color-coded value of a density function in an in-plane cross section of the molecular system. The function is calculated as the gradient norm of the electron density weighted by the scaled electron density.

The first 5 ps of the simulation are regarded as the equilibration stage. Polymer compression inevitably starts at the beginning of the time evolution and completes at 25 ps. The following 60 ps of trajectory are regarded as the equilibrated run. The timing of all analyzed events is zeroed at the beginning of the said production run, unless otherwise specified.

We employed a new kind of radial distribution function (RDF) in the analysis of the heteromaterial. Since the rGO layers are not perfectly parallel (or flat), the mathematical distance between them should be zero or undefined. However, in reality, the distance is measurable (with approximations). That motivated us to create a small Perl program which estimates approximate interlayer distance (R) RDFs in a few steps:

1. An arbitrary number of couples of stacked 6-atomic π–π rings are selected from neighboring rGO layers. Our selection was of 6 such couples for each interlayer distance.
2. The geometrical center (G) of the atoms in one of the rings is calculated.
3. The atoms of the remaining ring are split into two groups of three atoms each. One group is made of the odd-numbered (by chemical convention) atoms, while the other is of the even-numbered atoms. Since each group lies in exactly one plane, the classical equation (Equation (1)) of that plane is solved. In Equation (1), A, B, and C are the coefficients of the x, y, and z coordinates of the plane. A, B, and C also form the normal vector of that plane. D is the constant term in the equation.
  (1) Ax+By+Cz+D=0
4. From the two equations, A, B, and C are averaged into a new, single equation. This represents the “plane” of the second ring (in each ring couple).
5. The distance between G and the “plane” of the second ring is regarded as the approximate distance between the two rings. It is calculated by the classical equation for the distance between a point and a plane (Equation (2)). In that equation, x_0_, y_0_ and z_0_ are the coordinates of the point.
  (2) $$R=\left|Ax_{0}+By_{0}+Cz_{0}\right|/\sqrt{A^{2}+B^{2}+C^{2}}$$
6. The distances between all ring couples are averaged for each frame of the trajectory. The resultant number is considered as the approximate interlayer distance and it is used to calculate the corresponding RDF.

## 3. Results and Discussion

### 3.1. Structure and Strategy

**Figure 1:**
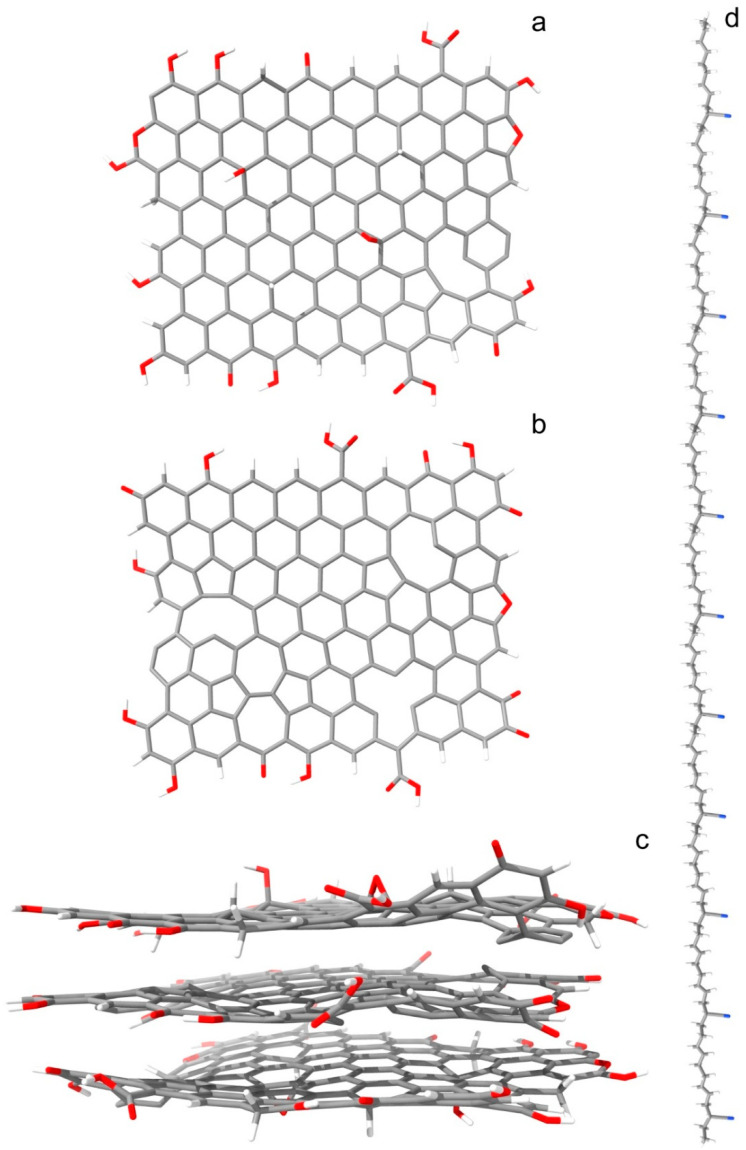
Optimized rGO and PBDAN structures (before the dynamical simulations) are presented: (**a**) the top layer of the rGO, which is identical with the bottom one, is shown; (**b**) the middle layer of the rGO; (**c**) the entire rGO system; (**d**) a single PBDAN chain. Color coding: H—white, C—gray, N—blue, O—red.

The studied heteromaterial consists of a flake of three-layered reduced graphene oxide, surrounded by multiple chains of butadiene acrylonitrile copolymer. The flake consists of 548 atoms (Figure 1). Of these, 409 are carbon atoms, 55 are oxygen, and 82 are hydrogen. The oxygen content is 12% (ignoring the hydrogen), corresponding to the reduced graphene oxide content. The top layer contains two types of in-plane defects: five carbon atoms are in the sp^3^ state of hybridization, while one sp^2^ hybridized carbon is missing (atom vacancy). Two of the sp^3^ carbons are neighbors, bound to the same oxygen atom (forming an epoxy group). The bottom layer is a replica of the top layer. The in-plane defects in the middle layer comprise three atom vacancies and a Stone–Wales defect. The dimensions of the rGO nanoparticle (largest nucleus to nucleus distances) are 23.46 × 21.45 × 10.83 Å. The size of the explicit QM cell is 30 × 30 × 30 Å.

Each polymer chain consists of 22 butadiene (BD) and 11 acrylonitrile (AN) monomers in an even distribution. Within this, each macromolecule contains 309 atoms. The simulation includes 48 chains. Each has an initial length of 129.91 Å, according to nuclear positions. In the initial system, they are arranged parallel to each other and to the rGO flake (Figure S1). The maximum transverse radii of the BD and AN fragments are 3.08 and 4.53 Å, respectively. The surface area of the substrate can accommodate multiple PBDAN chains. The total number of atoms in the modeled heterosystem is 15,188. The size of the periodic box at the beginning of the dynamical simulation is 140 × 150 × 120 Å.

The experimental copolymerization of butadiene and acrylonitrile typically results in tangled, creased chains, as the monomers are in a fluid state. To achieve such geometry, we positioned the polymer macromolecules at an increased distance (≥15 Å) from each other. During the system-compression stage of the NPT dynamics, the PBDAN chains spontaneously crease and entangle, while the large distance between them is brought to a realistic minimum.

### 3.2. Geometry and Energy

**Figure 2:**
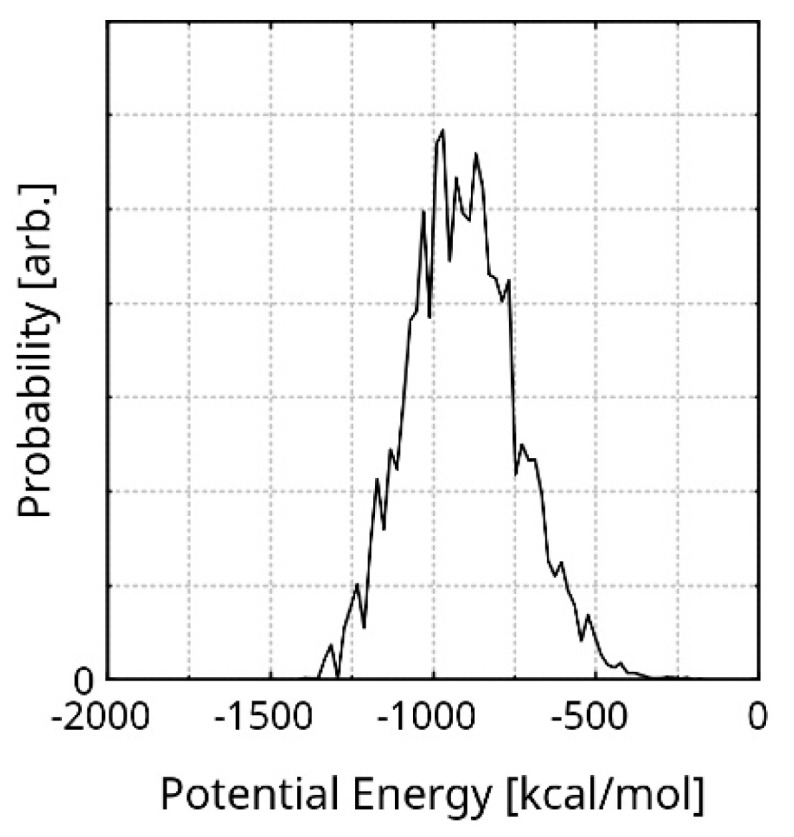
Probability distribution of the potential energy, during the equilibrated run. The X axis is zeroed at the highest energy in the equilibrated stage.

In order to select an exemplary geometry from the simulation trajectory, we took into account the presence of equilibrium fluctuations in the total energy (among other properties) and the principle of minimum energy. Additionally, the lowest value of the potential energy in the trajectory could be an artefact of the methods, and is realized only once. In order to find the most probable potential energy of the system, we calculated the probability distribution of this quantity over the equilibrated stage (Figure 2). The peak of the diagram is at −901.07 kcal/mol (the X axis is zeroed at the highest energy of the equilibrated system). The Half-Width Half-Maximum (HWHM) of the distribution is −337.33 kcal/mol. The corresponding geometry is visualized in Figure S2. The dimensions of the rGO and the explicit cell are 25.72 × 23.28 × 11.53 Å and 58.20 × 62.36 × 49.89 Å, respectively. The stabilization of the initial system geometry to the exemplary one is 10,363 kcal/mol. This value is only an intrinsic measure of the system model. One of the reasons it is so large is the realistic size of the system. Another is the minimal number of weak interactions in the initial geometry (rGO-only), due to large intermolecular distances. Furthermore, a significant amount of stabilization arises from the creasing of the polymer chains, as their original (entirely linear) shape is rather unrealistic.

### 3.3. Basic Structure

After the equilibrated stage, although flexible, the copolymer phase did not exhibit any significant conformational changes or chain displacements.

Throughout the trajectory, the rGO layers remain stacked. This is expected, as the large conjugated π-systems of the rGO planes cause interlayer π–π stacking, similar to that in graphite. As we have noticed in our previous research [78,79], adsorption to graphene and similar materials is particularly exothermic and results in significant stabilization. The energetic effect of such a process is comparable to the barriers of typical organic reactions. Nevertheless, a slight parallel displacement of the bottom rGO layer occurs between 1.0 and 4.7 ps of the compression stage (not the equilibrated run). Additionally, a slight rotation of the top layer is apparent between 6.0 and 7.9 ps of the compression stage. The polymer chains never enter the interlayer space of the rGO.

**Figure 3:**
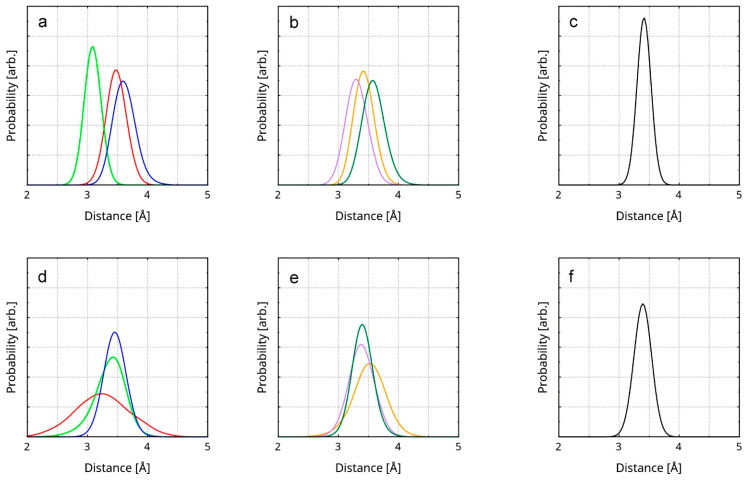
RDFs of the interlayer distances in the rGO are presented as follows. Each RDF is constructed over the averaged distance between 6 ring pairs. (**a**) RDFs of the distances between 3 of the ring pairs between the top and the middle layer; (**b**) RDFs of the distances between the remaining 3 ring pairs between the top and the middle layer; (**c**) RDF of the averaged distance between all 6 ring pairs; (**d**) RDFs of the distances between 3 of the ring pairs between the middle and the bottom layer; (**e**) RDFs of the distances between the remaining 3 ring pairs between the middle and the bottom layer; (**f**) RDF of the averaged distance between all 6 ring pairs. The RDFs of the ring pairs are represented by colored lines. The interlayer distance RDFs are represented by black lines. The selected ring pairs are visualized in Figure S3.

The probability of the average interlayer distance between the top and middle layer peaks at 3.41 Å (Figure 3). The corresponding value for the bottom and the middle layer is 3.38 Å. The reason why the second distance is slightly smaller is most probably the spontaneous occurrence of covalent bonding between the involved layers (all reactions are discussed in Section 3.5). Both values correspond very well to the experimental result for rGO (3.50 Å [80]). The accordance to the experiment validates the accuracy of the computational model. It also substantiates the precision and methodology in the estimation of the interlayer distance RDFs. Additionally, the result provides further confirmation of the reason why the rGO layers remain stacked throughout the trajectory—the short interlayer distance means a large stabilizing effect of the cumulative, whole-layer π–π stacking. As expected, the interlayer spacing in rGO is very close to that in the structure of graphite (3.34 Å [81]). Since there are no foreign atoms between the carbon planes, the distribution of the π density is only altered by the in-layer defects and the edge-bound substituents.

There are two topological patterns in the adsorption of the polymer chains on the substrate—trains and loops. The dominant type is trains. As expected, the powerful vdW forces near the surface of graphene-like materials dominate the dispersion forces around the polymer chains. The PBDAN chains, which are initially linear and isotactic, undergo folding and entanglement during the compression. This causes a complete loss of both long- and short-range symmetry.

### 3.4. Intermolecular Bonding

**Figure 4:**
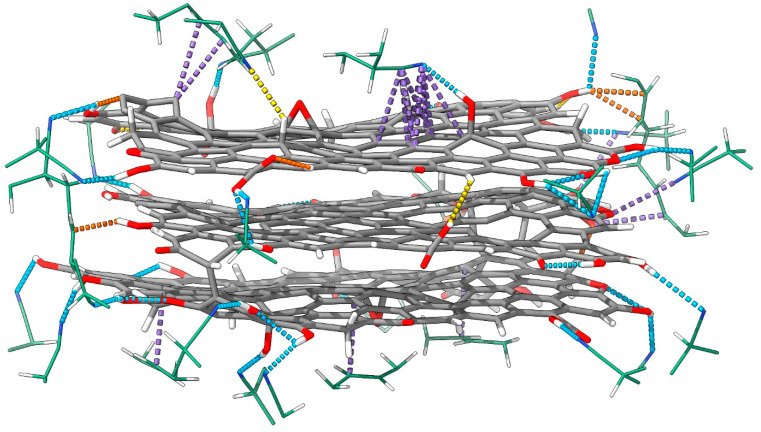
The geometry of the rGO and the nearest PBDAN atoms in an equilibrated run frame is presented, exhibiting all intermolecular interactions in the border region. Color coding: H—white, C—gray, N—blue, O—red. Hbs are colored in blue, π–π stacking is shown in purple, σ–π stacking is in orange, and σ–n stacking is in yellow.

We found four types of non-covalent interactions within the reduced graphene oxide and the rGO/PBDAN border region: hydrogen bonds (Hbs), π–π stacking, σ–π stacking, and σ–n stacking. While Hbs and π–π interactions are rather common, the last two types of intermolecular bonding both occur and are examined less often. A σ–π interaction (hyperconjugation, non-bonding resonance) occurs when an s-AO (usually in a sigma bond) acts as a donor of electronic density towards an empty or partially filled p-AO or π-MO. σ–n stacking may be realized when an electron-rich lone pair attracts a σ-hole (an electron-poor s AO in a sigma bond). While an exchange of electronic density is common in π–π interactions, σ–π and σ–n stacking are usually manifest through dispersion forces. Both of these weak, non-covalent interactions are a known source of stabilization in chemistry and play an important role in the geometry and spatial arrangement of large molecular systems [82]. Ordering such interactions by strength is not straightforward, as the energy contribution of each kind is highly variable. While an exchange of electronic density can occur through Hb (until a certain interatomic distance), other weak interactions are considered purely electrostatic. Typically, the strength of a hydrogen bond is between 1 and 40 kcal/mol [83]. Other non-covalent interactions can provide stabilization of 1 to 5 kcal/mol [84]. It is proposed that π–π stacking provides a larger reduction in energy than σ–π [72,85]. The dispersion interactions in the rGO and the rGO/PBDAN border region are visualized in Figure 4.

**Figure 5:**
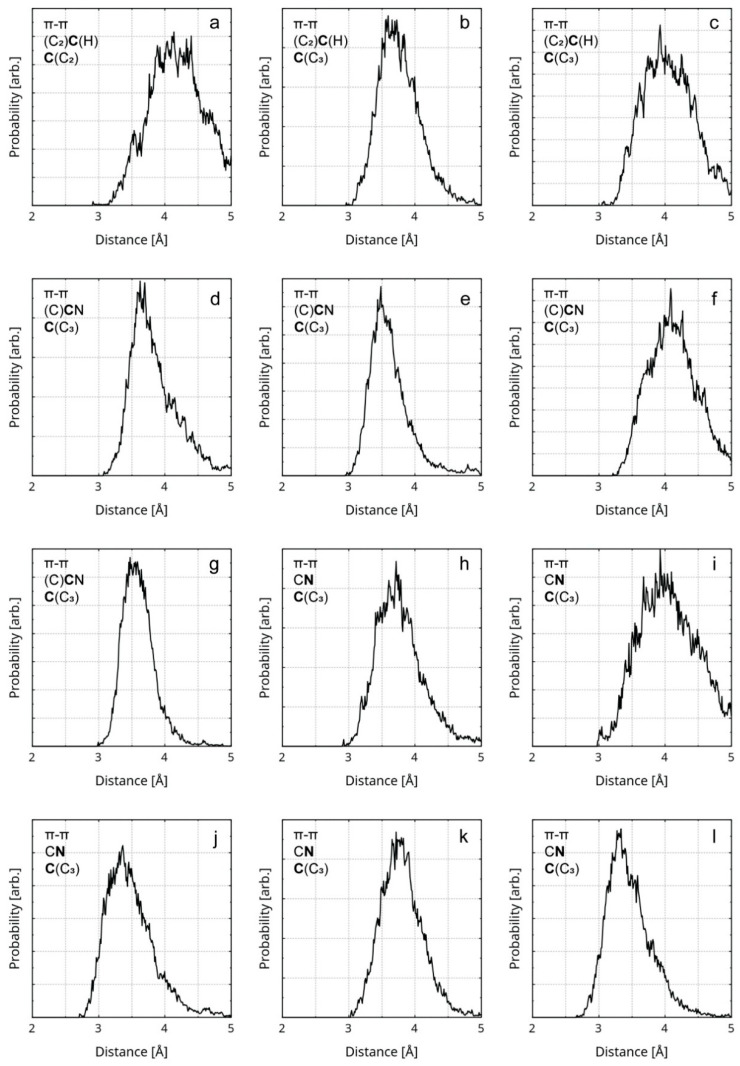
RDFs of the distances between the atoms (from the rGO and the polymer) participating in π–π stacking are presented. The first line of the legend in each graphic designates the interaction type. The second line in each legend designates the participating atom type of the polymer (in bold) and its covalent neighbors. The third line in each legend designates the participating atom type of the rGO (in bold) and its covalent neighbors. The diagrams represent the instances of π–π stacking between the following atom pairs: (**a**) a butadiene C atom and an rGO radical sp^2^ C atom; (**b**,**c**) a butadiene C atom and an rGO sp^2^ C atom; (**d**,**e**) a nitrile C atom and an rGO sp^2^ C atom; (**f**,**g**) a nitrile C atom and an rGO sp^2^ C atom; (**h**–**l**) a nitrile N atom and an rGO sp^2^ C atom.

**Figure 6:**
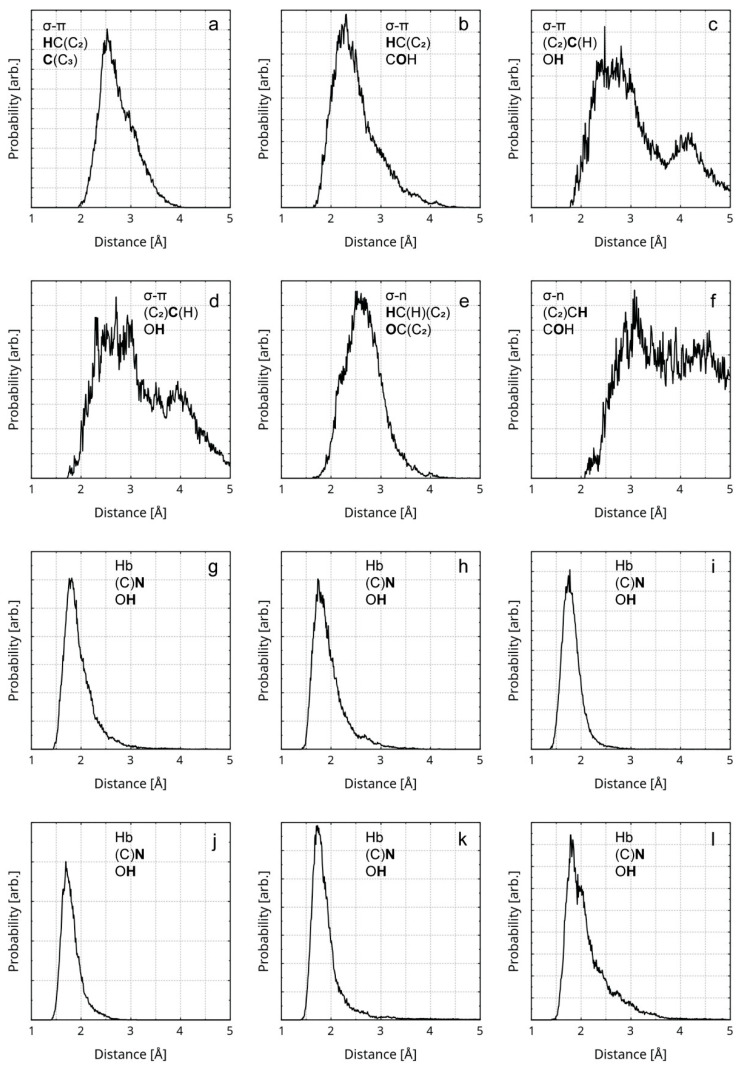
RDFs of the distances between atoms participating in σ–π stacking, σ–n stacking, and Hbs are presented. The first line of the legend in each graphic designates the type of interaction. The second and the third line designate the participating atom type (in bold) and its covalent neighbors. All diagrams represent rGO–polymer interactions, except for (**a**,**e**,**f**), which represent rGO interlayer interactions. The diagrams represent the following instances of weak interactions: (**a**) a σ–π stacking between a H atom and an sp^2^ C atom; (**b**) a σ–π stacking between a H atom and a hydroxyl O atom; (**c**,**d**) a σ–π stacking between an sp^2^ C atom and a hydroxyl H atom; (**e**) a σ–n stacking between a methylene H atom and a quinoid O atom; (**f**) a σ–n stacking between an aromatic H atom and a hydroxyl O atom; (**g**–**l**) a hydrogen bond between a nitrile N atom and a hydroxyl H atom.

There are 16 weak interactions within the structure of the reduced graphene oxide. These comprise 10 hydrogen bonds, 3 counts of σ–π stacking, and 3 counts of σ–n stacking. The only rGO-specific π–π interaction is the interlayer stacking. RDFs of the corresponding interatomic distances can be found in Figure 5, Figure 6, Figures S4 and S5.

Within the rGO/PBDAN adsorption region, there are a total of 42 non-covalent interactions. These comprise 20 hydrogen bonds, 17 counts of π–π stacking, 4 counts of σ–π stacking, and 1 count of σ–n stacking. Together, those weak attractions (Figure 4) provide a significant energetic stabilization in the heterogenous material. Additionally, they preserve the position of the rGO particle within the polymer phase. π–π stacking for the interatomic distance RDFs, including rGO/PBDAN, is shown in Figure 5. The remainder are shown in Figures S4 and S5.

As expected, interphase stabilization is reciprocal to the content of unsaturated polymer fragments.

**Figure 7:**
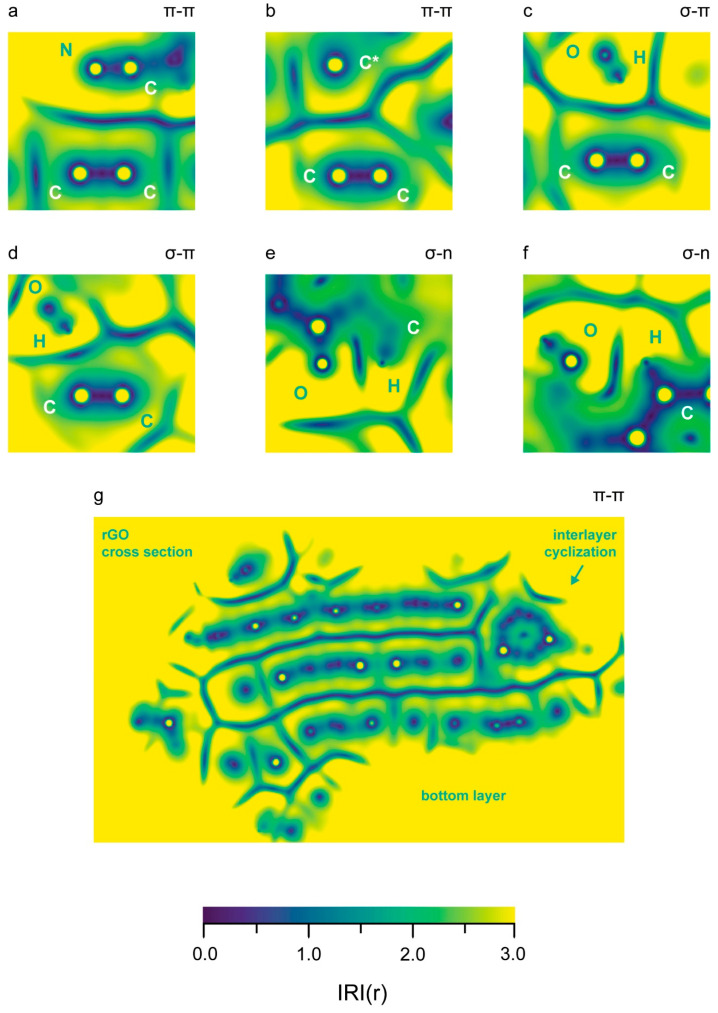
Visualization (in-plane IRI diagrams) of intermolecular interactions between the rGO phase and the polymer chains is presented. All images correspond to interactions in the trajectory frame, visualized in Figure 4. Higher IRI values are represented in yellow. (**a**) A set of 4 counts of π–π stacking: each atom of the –CN group stacks with both C atoms from the rGO; (**b**) π–π stacking between a radical C atom in the rGO and an vinyl group in the PBDAN; (**c**,**d**) σ–π stacking between the H atom of a –OH substituent and a vinyl group in the polymer; (**e**) σ–n stacking between an aromatic H atom and quinoid O atom; (**f**) σ–n stacking between an aromatic H atom and the O atom of an aromatic –OH group; (**g**) a complete cross section of the rGO, displaying the interlayer π–π stacking. The bottom layer is down; the interlayer cyclization is in the top-right; and a few non-covalent interactions are also visible.

Two of the rGO atoms involved in intermolecular bonds neighbor a vacancy defect. One of the corresponding distance RDFs is shown in Figure 5a, while the other is shown in Figure S4l. Some σ–π and σ–n interactions are indicated between the rGO layers. The RDFs of the distances corresponding to these intermolecular bonds are represented by diagrams (a), (e), and (f), in Figure 6. The maximum length of a Hb is considered to be 4.0 Å [86]. It is accepted that the typical distance for π–π stacking is between 3.0 Å and 4.0 Å, with 3.5 Å being optimal [87,88]. The upper limit of the distance for σ–π [89,90] and σ–n [91,92] dispersion interactions is considered to be the sum of the van der Waals radii of the atomic pair: 2.9 Å for a H-C, 2.75 Å for H-N, and 2.72 Å for H-O. When employing these numbers, only the interlayer π–π stacking within the rGO remains throughout the trajectory. The rest of the non-covalent interactions tend to occasionally vanish during the equilibrated stage of the trajectory. The weakest and most exotic type—σ–n stacking—is also the least persistent (Figure S6d). A select few of the weak interactions are visualized in Figure 7, through in-plane IRI analysis. The employed geometry is displayed in Figure 4. The exact zones and degree of intermolecular density overlap are an additional proof of non-covalent binding. Visually, the volume of the interactions appears to rise in the following order: σ–n < σ–π < π–π. The IRI values of the maxima π–π and σ–n interactions appear very similar to the values of the innermost (closest to the nuclei) portions of the valent electronic density. It seems that σ–π interactions possess the lowest strength, perhaps due to the fact that the σ–n distances are natively constrained to smaller values by being intramolecular.

The abundance of conjugated π-density in rGO is the reason behind its high potential, both as an acceptor and as a donor of electrons. Its relatively low electrical resistance enables the material to serve as a conductor between electron-deficient and electron-rich molecular fragments. This only elevates the material’s potential for intermolecular bonding.

On a fundamental level, the properties of the matter are an expression of the spatial distribution of electronic density and nuclei. In other words, the distinguishing characteristics of different states of matter, alongside specific characteristics of any given material, arise from the strength of interatomic bonding, and the sign and strength of intermolecular interactions. Explicitly, mechanical properties, such as Young’s modulus, bulk modulus, shear modulus, and tensile strength, are proportionate to resistance to intermolecular displacement (heteromaterial stabilization). The same applies to thermal durability. Hooke’s law, Poisson’s ratio, and storage modulus are also related to the physical properties of the chemical structure. In simple terms, in order to draw conclusions regarding the enhancing properties of reduced graphene oxide towards the polymer matrix, we need to understand the structure of the two phases and the interactions within the adsorption region.

The commonly known elasticity of acrylonitrile rubber can be explained on the basis of three factors: (1) the flexible carbon frame of the polymer chains, (2) the lack of covalent bonding between them, and (3) the apparent high degree of entanglement between the said PBDAN macromolecules (Figure S2). Due to the same factors, it is reasonable to classify such polymers as overcooled liquids.

Hydrogen bonds and stacking do not exist within the PBDAN phase; there are no polar hydrogens for the former and a very low spatial chance, combined with no electrostatic reasons for the latter. Moreover, the rGO particles possess strong, covalent bonding throughout the layers, combined with a significant interplane stabilization. The abundance of attractive intermolecular interactions between the two phases results in a new material with significantly increased constitution, which can only mean an improvement in mechanical and thermal properties across the board. Increased rGO concentrations may increase the thermal and electrical conductivity of the polymer, which is an insulator. However, the elasticity can only be preserved below a certain threshold of concentration of the stiffening rGO. The following factors can serve as a measure of the interphase binding: (1) the large energetic effect of polymer creasing and adsorption, and (2) the proportion (0.033) between the HWHM of the energy probability during the equilibrated stage and the total stabilization of the polymer-binding process.

**Figure 8:**
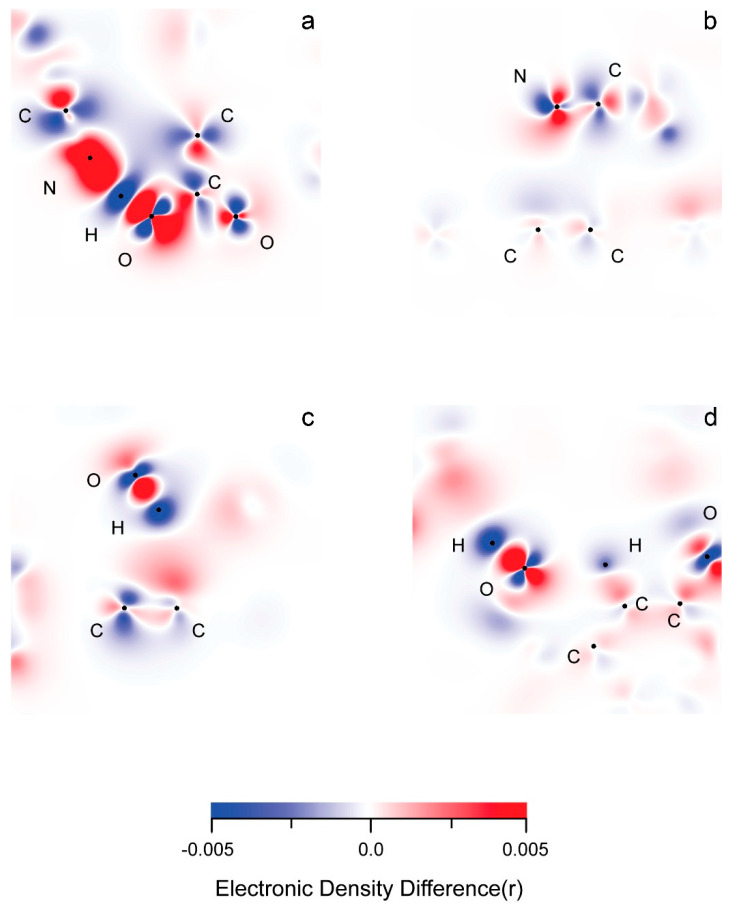
Electronic Density Difference plots of different bonds and structures are presented as follows: (**a**) hydrogen bonding; (**b**) π–π stacking in Figure 7a; (**c**), σ–π stacking in Figure 7c; (**d**) σ–n stacking in Figure 7f. Increasea in electronic density are represented in red.

In rGO, the electronic density (ED) gain surpasses the loss (Figures S7 and S8). The opposite is true for the polymer chains. The ED changes in the middle substrate layer are minimal and mostly evident in zones of near non-covalent interactions involving its functional groups. As expected, there is an obvious delocalization of density changes within the carbon framework of the rGO layers. The layer-wide π-conjugation can easily explain this. It should also be responsible for the evidently smaller total, local ED alteration within graphene fragments involved in intermolecular interactions (Figure 8, Figures S7 and S8).

More often than not, there is asymmetric, atom-by-atom alternation of density gain and loss in aromatic rings.

The atom types can be placed in the following categories of charge alteration:

(1) Donors in every case: aliphatic H of the polymer.
(2) Mostly donors in every case: ether O.
(3) Mostly acceptors in every case: epoxy O, carboxyl carbonyl O, carboxyl hydroxyl O.
(4) Donor in most cases: nitrile C, hydroxyl H.
(5) Acceptors in most cases: quinoid O, aromatic C.
(6) Gain and lose density in similar amounts: aliphatic C of the polymer.
(7) Gain or lose in a similar number of cases: aromatic H, hydroxyl O.

The donor/acceptor trends of specific atom types represent the stabilizing effects of adsorption on a basic level. The results can contribute to the explanation behind the properties of ligand–substrate systems. Additionally, they can facilitate the rational design of heteromaterials involving the investigated functional groups.

As expected within intermolecular interactions involving electronic systems with opposite charges, the atoms which are directly involved undergo density changes with a reverse sign (Figure 8 and Figures S7–S9). There appears to be an alternation in gain (+) and loss (−) of ED within the structure of functional groups participating in non-covalent interactions with a single atom. An example of such an alternation is designated by AO type in Figure S9 (hydrogen bonding). Because of the self-repulsion of the electronic density, a gain in one molecular orbital would provoke loss in a neighboring one, and vice versa. Occasionally, neighboring MOs overlap to form one electronic cloud, regardless of their spatial symmetry, just as long as they are all either donors or acceptors (Figure S8).

### 3.5. Reactions

**Figure 9:**
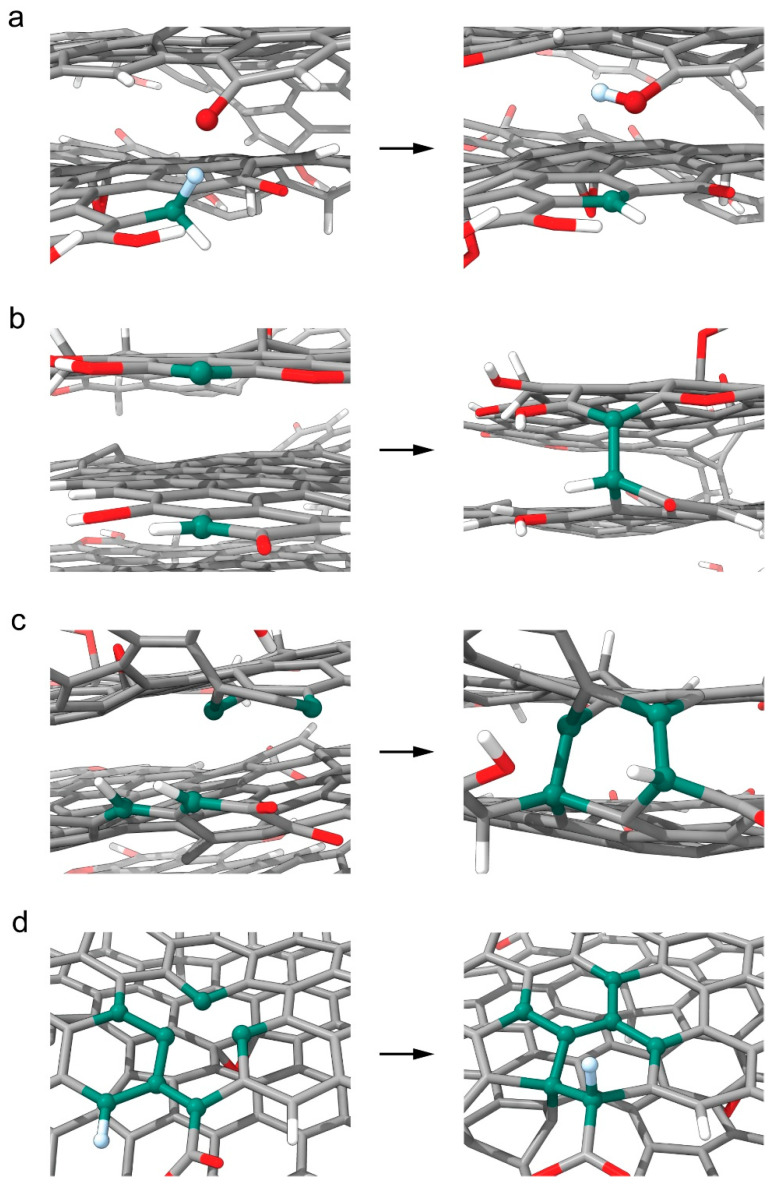
Reagent and final product geometries of the rGO fragments which participated in spontaneous reactions are presented as follows: (**a**) interlayer (middle to top) hydrogen transfer, converting a keto group in a hydroxyl group; (**b**) formation of interlayer (middle, top) covalent bond; (**c**) interlayer (middle, top), two-stage cyclization; and (**d**) (middle layer) ring reformation/self-repair reactions (due to a vacancy defect), accompanied by a hydrogen transfer. Reactive atoms are visualized as spheres. H atoms are shown in white. Reactive H atoms are shown in light blue. C atoms are shown in gray, reactive C atoms in blue-green, O atoms in red, and reactive O atoms in darker red.

Several (9) spontaneous reactions occurred during the equilibration stage. One of them was a hydrogen transfer between a methylene group in the rGO middle layer and a quinone group a spart of the π-system of the top layer (Figure 9a). This occurred at 2959 fs during a slide-shift between the top and middle rGO planes, which brought the reactive substituents together. The process resulted in the creation of one methine group and one hydroxyl group. Both were conjugated to their respective layers. In previous work, we had already investigated the surface chemistry of rGO—more specifically, various H-atom transfer reactions [66].

The simulation enabled the appearance of multiple covalent C-C bonds between neighboring layers. The first such reaction occurred at 3821 fs (Figure 9b). The second bond was formed at 8556 fs, and the last one at 16,437 fs (Figure 9c). The first and the second interlayer coupling can be viewed as two halves of a process of cyclization—the resulting fragment is a six-membered ring system with no heteroatoms. Two of the atoms in the ring remained in (mostly) the sp^2^ state, while the rest assumed sp^3^ hybridization. In reality, the hybridization state is never perfect/singular. Covalent interlayer bonding prevents layer shifting and unstacking. The result is inertness to changes in intermolecular distance and orientation of polymer chains, bound to different rGO layers. Hence, it is expected that the covalent interlayer bonding improves the properties, regarding material toughness.

The second (and last) hydrogen transfer is between a methanetriyl and a methylidene radical functionalized with a carboxyl group. The radical is a part of the π-system of the middle rGO layer. The single electron cannot conjugate for reasons of orbital symmetry and is therefore highly reactive. The reaction is concomitant to the process, which we view next and transpires within the same molecular fragment. A ring opening reaction within that fragment results in the radical reagent and provokes the H-transfer. Both substituents are covalently bound to the same neighboring C atom. The products are a methanetriyl radical and a functionalized methylidene group (Figure S10a). It is to be noted that, for reasons of symmetry, the orbital containing the radical electron of the radical product is partially conjugated to the π-electronic system of the rGO layer. The carboxyl substituent remains bound to the second participating functional group throughout the simulation. The reaction occurs at 13,510 fs. Immediately after, the H atom and the H-donatingcarbon remain stacked through a σ–σ interaction, due to orbital orientation andproximity, as the corresponding distance is ~1.9 Å (Figure S10a). During the concomitant process, the acceptor changed its hybridization state from sp^2^ to sp^3^. The reverse is true for the donor. The two atoms are then bound together (Figure 9d).

The remaining reactions represent a process of self-repair—a spontaneous stabilization of a vacancy defect, within the carbon framework of the rGO. In its essence, it is a process of reforming of condensed rings, consisting of multiple reactions of ring opening and cyclization. No heteroatoms are involved. Initially, the reactive fragment contains a six-membered ring, which shares two of its bonds with two out of three condensed rings, missing a common C atom (Figure 9d). In other words, it is a system consisting of one six-membered ring, condensed to 13-membered annulene, both of which are integrated within the C frame of the middle rGO layer. This process occurs in the vicinity of the described interlayer cyclization, as the six-membered ring participates in both. A total of five reactions occur: 2 for C-C bond cleavage and three for C-C bond formation (Figure S10b). Since the rings are unsaturated and a part of the rGO layer’s π-electronic system, the said bonds have a partial double character. The self-repair results in four condensed rings: one five-membered ring and three six-membered rings (Figure 9d). Effectively, the one-atom vacancy defect has vanished from the carbon frame of the rGO layer by moving towards the edge. One of the two-edge rings is composed of five atoms; this is because there is no source for C atoms to completely compensate for the vacancy. The process as described here begins at 676 fs and finishes at 14,636 fs. One of the carbons in the reactive fragment is the atom donor from the previous hydrogen transfer. Another is the acceptor. Only those two atoms assume sp^3^ hybridization. The donor also participates in the interlayer cyclization.

The process of self-repair shows a tendency towards the long-term stability of the rGO; instead of decay, and by inheritance, it also ensures the durability of the heteromaterial.

**Figure 10:**
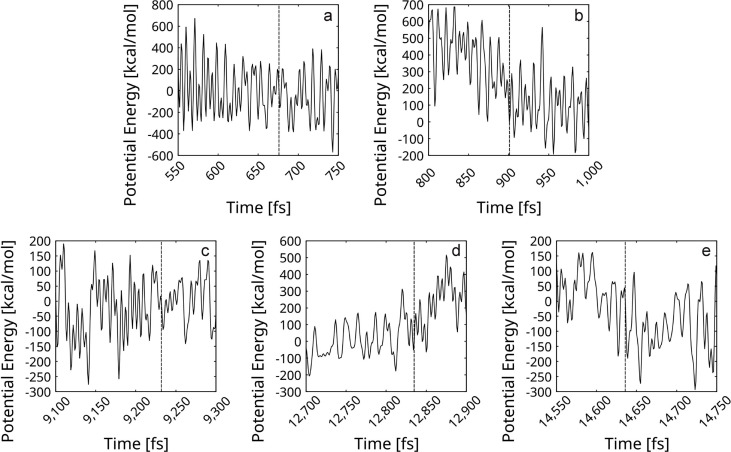
Potential energy profiles of the reactions of self-repair of graphene fragments within the rGO are presented. In each diagram, the ordinate is zeroed at the energy of the frame containing the corresponding transition state (TS). The frames of the transition states are designated with vertical dashed lines. The energy profiles belong to reactions, which saddle points occur at the following frames: (**a**) 676 fs; (**b**) 901 fs; (**c**) 9232 fs; (**d**) 12,835 fs; and (**e**) 14,636 fs.

As evident in Figure 10, all five reactions of self-healing occur without a noticeable barrier (most likely the barrier is not evident due to the energy fluctuations of the total system). In the light of this result, the overall process appears probable. The model corresponds well to the mentioned experimental report of spontaneous defect mending at room temperature and under reduced pressure [41].

Although the reaction occurred within the first 25 ps of the simulation, the rGO nanoparticle is probably equilibrated due to the following: (1) it consists of only 3.61% of the atoms in the whole system, (2) the rigid covalent framework of the rGO, and (3) the interlayer distance is equipoised very quickly, due to the realistic initial value and the strong intermolecular forces at the rGO surfaces. After all, the long equilibration time was chosen only due to the non-bonding interactions within the polymer phase and its size. Additionally, not one of the reactions occurs on the outer surfaces of the substrate, and the influence of the PBDAN is truly negligible.

Not a single atom left the rGO phase throughout the entire simulation.

## 4. Conclusions

The nanoscopic folding and binding of the copolymers, consisting of butadiene and acrylonitrile in a ratio of 2:1, of three-layered reduced graphene oxide were investigated using QM/MM Born–Oppenheimer Molecular Dynamics. The theoretical model mirrors the pressure and temperature conditions of a parallel experimental study in its final stages.

Throughout the trajectory, the layers of the rGO do not unstack and the polymer does not enter the space between them. The average interlayer distance is in good accordance with the experimental values. The rGO planes do slightly shift, relative to each other.

The compression/adsorption stage of the simulation yields highly creased and entangled polymer chains. The evident elasticity of such polymers arises from the flexibility of the σ-density carbon framework and the lack of covalent bonding between the chains.

Dozens of non-covalent intermolecular interactions were apparent during the ab initio dynamics. All were characterized by type and length probability. Four kinds of weak interactions were found: hydrogen bonds, π–π stacking, σ–π stacking, and σ–n stacking. In addition to the whole-layer stacking, 16 instances of such interactions existed for the rGO itself: 10 were hydrogen bonds, 3 involved σ–π stacking, and 3 involved σ–n stacking. The interface region between the two phases contained 42 additional weak bonds: 20 hydrogen bonds, 17 π–π interactions, 4 σ–π interactions, and 1 σ–n interaction. According to the RDF of each non-covalent bond, they would occasionally vanish in the trajectory of the equilibrated system. The small number of weak bonds involving σ-density indicates insufficient interphase stabilization with saturated polymer structures. However, as expected, the binding between the two phases is significantly stronger for ligands with π-MOs. Electronic density difference plots reveal that the carbon framework of the rGO layers mostly acts as an acceptor. The opposite is true for the polymer matrix. Density changes in the middle layer are mostly negligible and localized in areas of heteroatoms, participating in intermolecular interactions. Due to system-wide conjugation, adsorption induces charge changes throughout the carbon network of the rGO layers. Nitrile nitrogens and hydroxyl hydrogens tend to be donors. The aliphatic hydrogens of the polymer and the ether oxygens always lose density. Quinoid O atoms and aromatic C atoms are more often than not acceptors. The following atom types are always acceptors: epoxy O, carboxyl carbonyl O, carboxyl hydroxyl O. Aliphatic carbons in the polymer seem to gain and lose density in similar amounts. Aromatic H atoms and hydroxyl O atoms appear to be acting as either donors or acceptors in an even number of cases. In the intermolecular interactions with a σ-character, there is a clear gain/loss alternation over the structure of neighboring AOs of the participating functional groups. The abundant number of stabilizing intermolecular interactions provides a significant attraction between the rGO and the adsorbed PBDAN chains, which are, in turn, entangled in the rest of the polymer network. Such significant overall binding is the explanation behind the enhancing properties of the substrate. Resistance to intermolecular displacement means sustainability in structure. Among the effects is an improvement in mechanical properties, such as tensile strength, shear, bulk, and Young’s modulus. Another result is an increase in thermal durability. A good indicator for material stability (including interphase binding) is large whole-system stabilization, indicated here as 10,363 kcal/mol. Furthermore, the proportion between the HWHM of the energy probability distribution for the equilibrated system and the total stabilization is rather small, at 0.033.

Multiple (nine) reactions occurred during the simulation phase. Two of them were hydrogen transfer. One aliphatic H atom jumped to the oxygen of a quinoid group in another layer. Another (aromatic) H atom jumped to a functionalized methylidene radical. The radical was present due to a bond cleavage in an ongoing concomitant process in the same fragment. Three reactions resulted in single C–C covalent bonds, binding the top and the middle layer. Together, two of them represent intermolecular cyclization to a six-membered ring. Covalent bond formation between the layers prevents their shifting and unstacking. Such processes effectively contribute to the preservation of intermolecular distance and orientation between polymer chains, adsorbed on different rGO layers. A sequence of reactions of ring opening and cyclization resulted in self-repair of a graphene fragment within the middle rGO layer. Effectively, a vacancy defect traveled to the edge of the macromolecule and disappeared. No heteroatom left the rGO phase.

## Data Availability

The original contributions presented in this study are included in the article/Supplementary Materials. Further inquiries can be directed to the corresponding author.

## Supplementary Materials

The following supporting information can be downloaded at: https://www.mdpi.com/article/10.3390/nano16020113/s1.
