# Supplementary material for: QM/MM Dynamics Study of the Augmenting Effects of Reduced Graphene Oxide Towards the Butadiene Acrylonitrile Copolymer Matrix and Self-Repair of the Enhancer
Source: Nanomaterials (Basel). 2026 Jan 15;16(2):113. doi: 10.3390/nano16020113 (PMC12844241; doi:10.3390/nano16020113)
Supplement: Supplementary file 1 [file nanomaterials-16-00113-s001.zip › nanomaterials-4035168-supplementary.pdf]

QM/MM Dynamics study of the augmenting effects of reduced graphene oxide towards butadiene acrylonitrile copolymer matrix and self-repair of the enhancer

SUPPORTING INFORMATION

Kalchevski, D. A.[1)]; Kolev, S. K.[1)]; Ivanov K.V.[1)]; Dimov, D.A.[1)]; Kostadinova, A.S.[2)]; Aleksandrov H.A.[3)]; Milenov, T. I.[1)]

[1)]"E. Djakov" Institute of Electronics, Bulgarian Academy of Sciences, 72 Tzarigradsko Chausee Blvd., 1784 Sofia, Bulgaria
[2)]Institute of Biophysics and Biomedical Engineering, Bulgarian Academy of Sciences, Sofia 1113, Bulgaria
[3)]Faculty of Chemistry and Pharmacy, Sofia University "St. Kliment Ohridski", 1 J. Bourchier Blvd., Sofia 1164, Bulgaria

Optimized rGO/PBDAN geometry 2
Final rGO/PBDAN geometry 3
Ring selection for the interlayer distance RDFs 4
Additional RDFs of the rGO – polymer system 5
Additional RDFs of the rGO – polymer system 6
Time-evolution of distances in intermolecular interactions 7
Top layer / polymer electronic density difference due to adsorption 9
Bottom layer / polymer electronic density difference due to adsorption 9
Alternation of the sign of the electronic density difference due to a hydrogen bond 10
Details in some rGO reaction 11

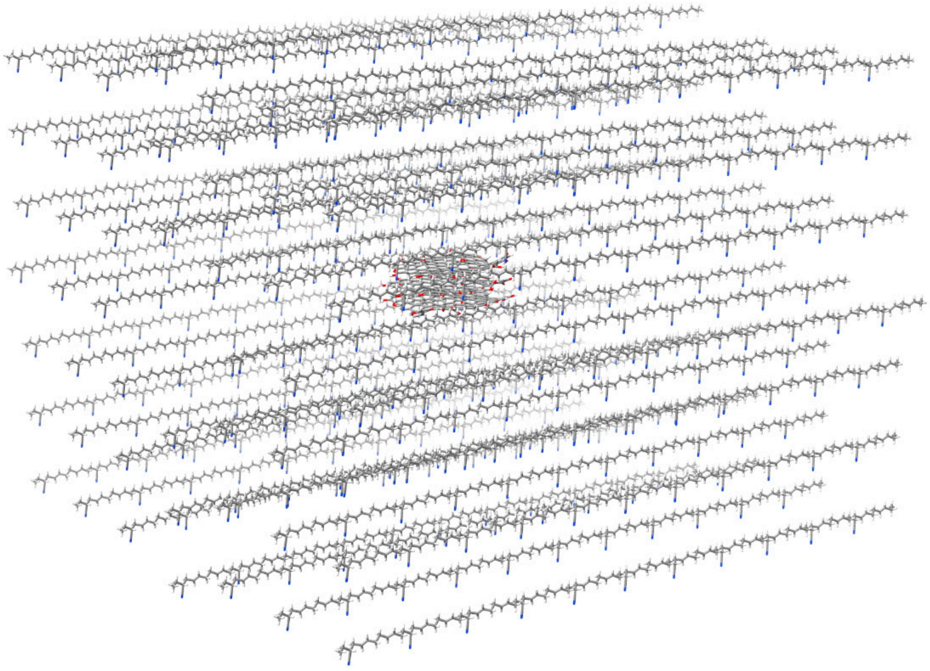

Figure S1: Optimized rGO/PBDAN geometry.

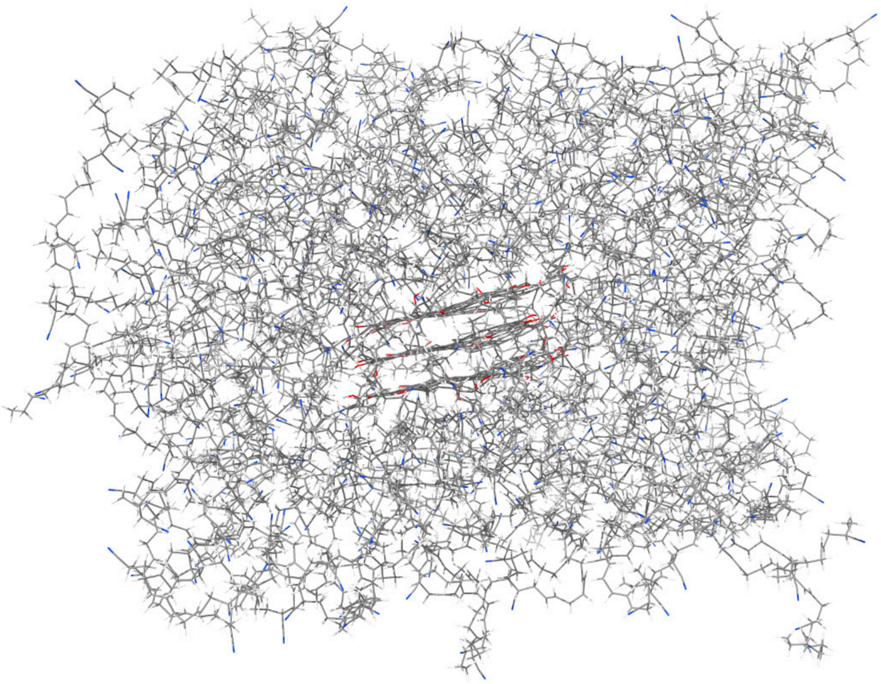

Figure S2: Frame of the equilibrated trajectory, corresponding best to the most probably potential energy of the rGO/PBDAN system.

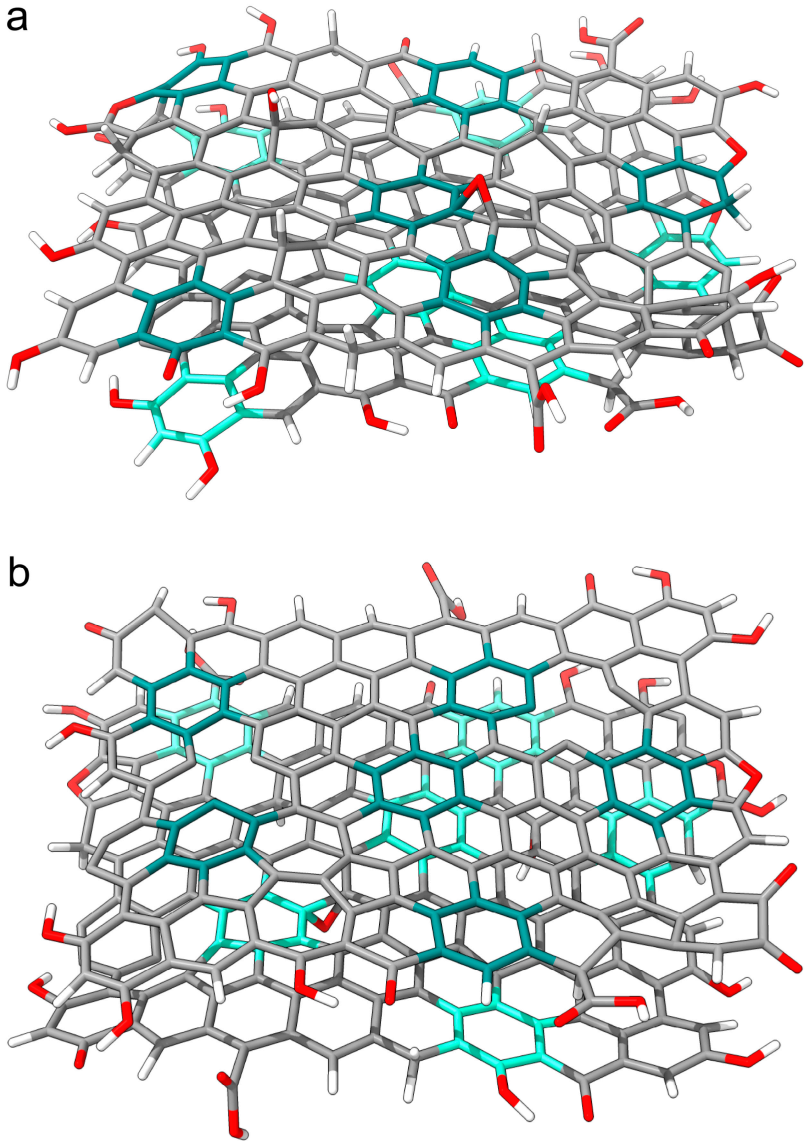

Figure S3: Ring fragment pairs, selected for calculating the interlayer RDFs: (a) in the top and the middle layer and (b) in the middle and the bottom layer.

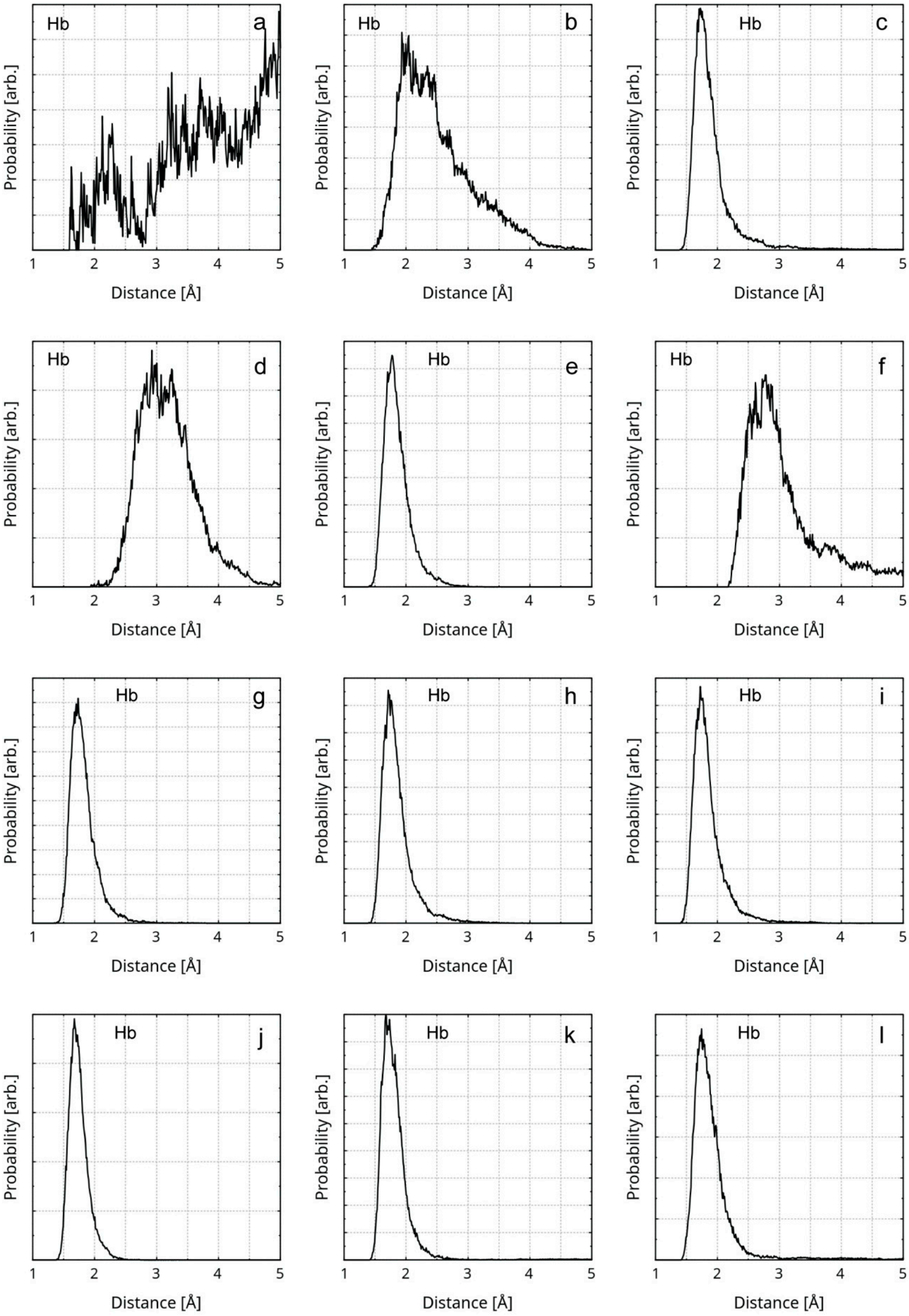

Figure S4: Additional RDFs of distances between atoms, participating in Hbs. All diagrams represent rGO – polymer interactions.

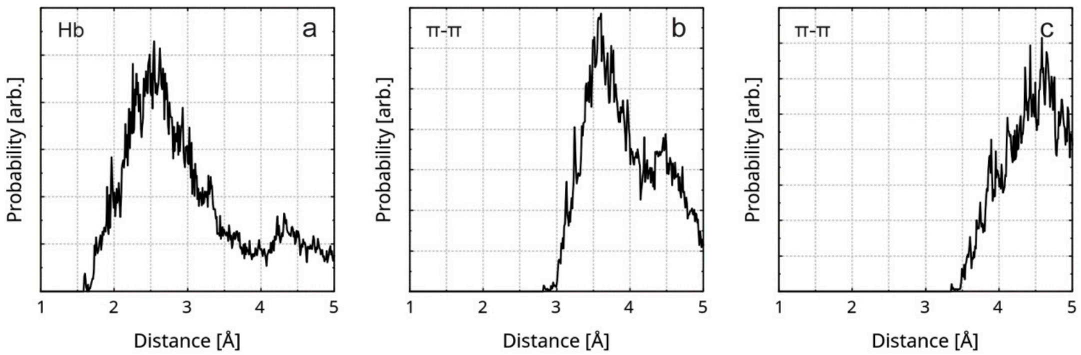

Figure S5: Additional RDFs of distances between atoms, participating in intermolecular interactions. All diagrams represent rGO – polymer interactions.

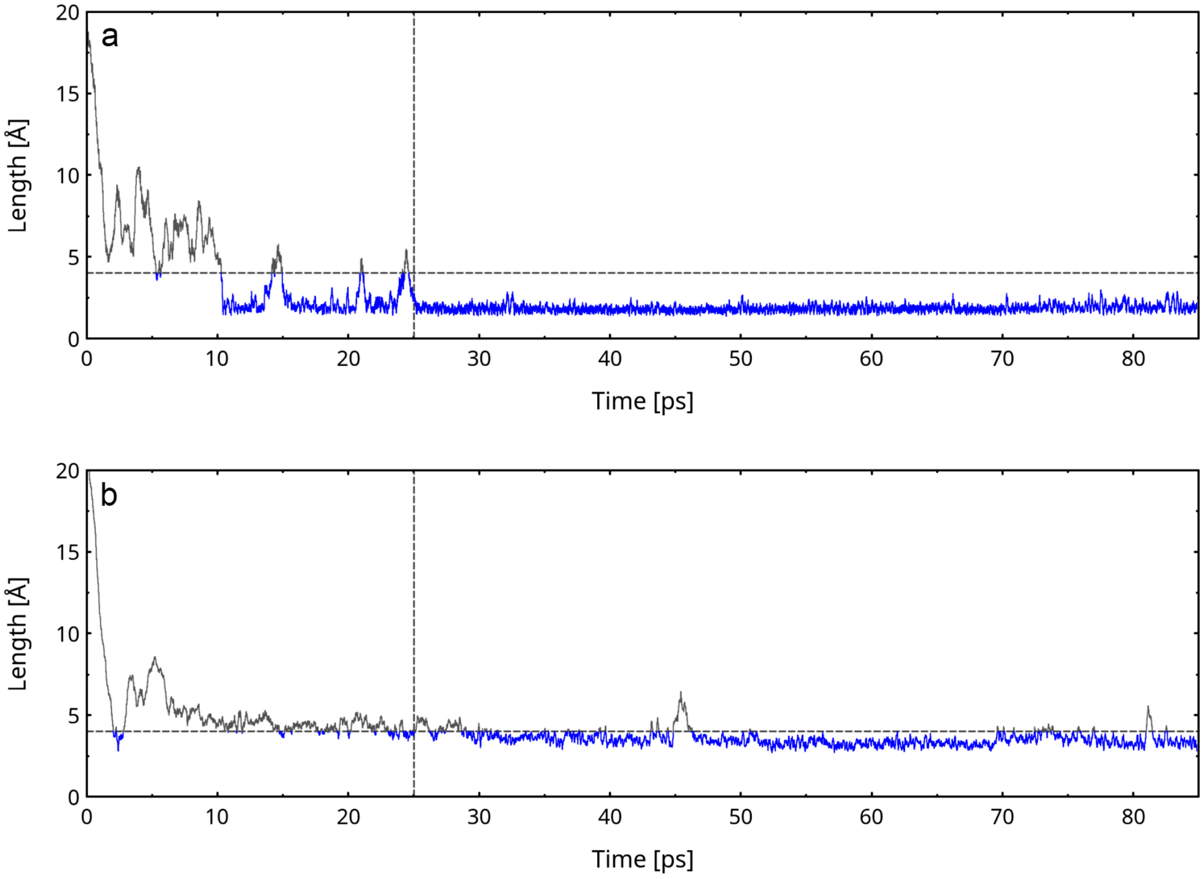
a
b
Length [Å]
Time [ps]
0
5
10
15
20
0
10
20
30
40
50
60
70
80

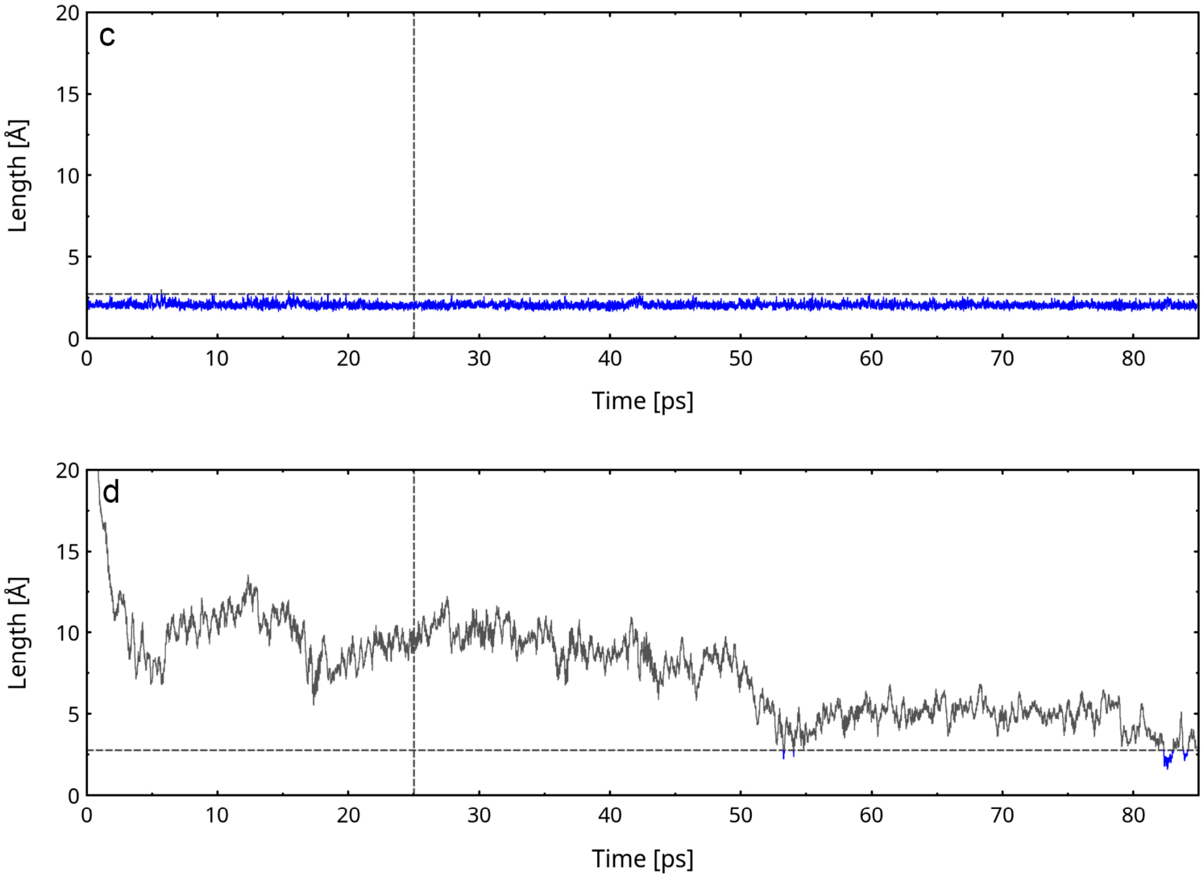

Figure S6: Time evolution of the distances in different intermolecular interactions. (a) the hydrogen bond in Fig. 8a. (b) the π-π stacking in Fig. 7a and 8b. (c) the σ-π stacking in Fig. 7c and 8c. (d) the σ-n stacking in Fig. 7f and 8c. The vertical dashed line represents the frame at the end of equilibration. The horizontal dashed line represents the threshold distance for the type of weak interaction. The distance value of the trajectory frames, in which there is an effective stacking, is in blue.

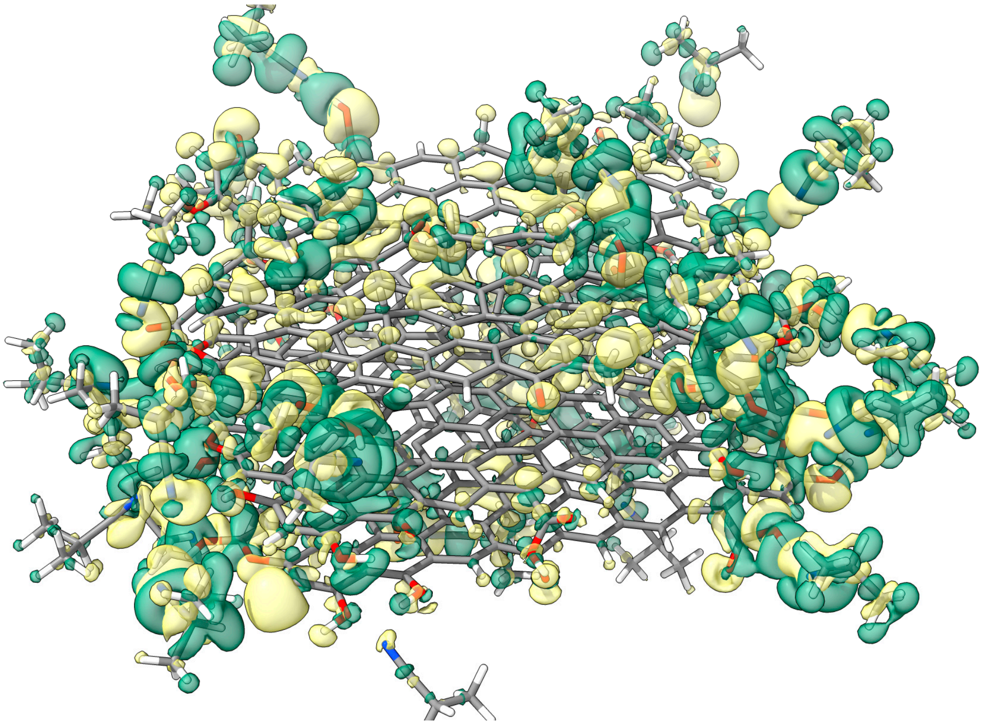

Figure S7: Changes of the electronic density because of adsorption to the top rGO layer. Loses are in green. Gains are in yellow.

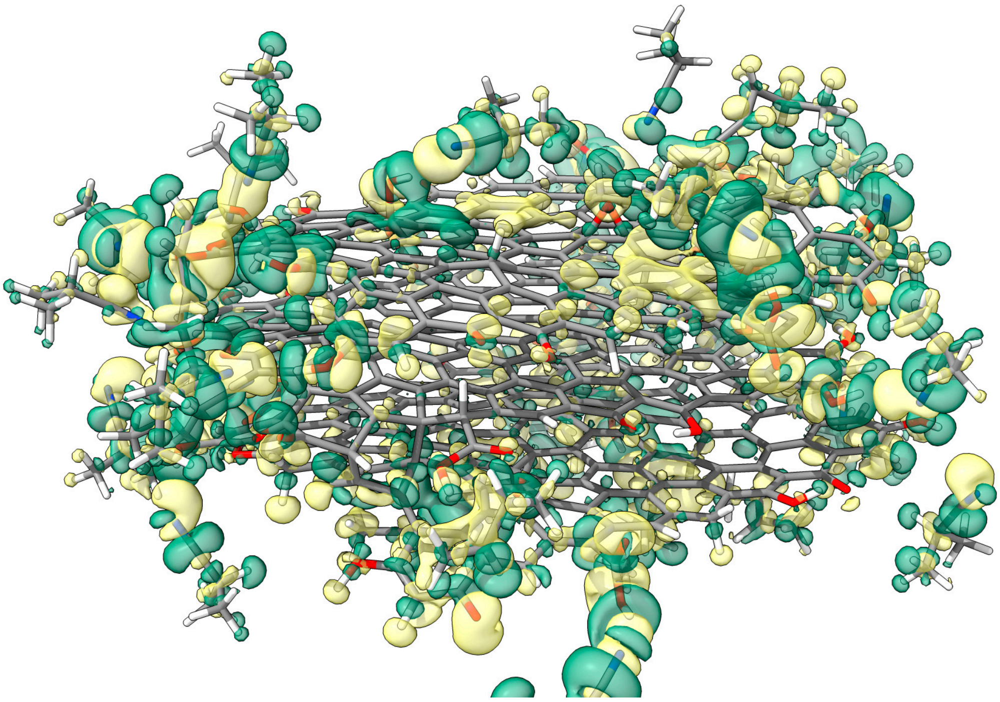

Figure S8: Changes of the electronic density because of adsorption to the bottom rGO layer. Loses are in green. Gains are in yellow.

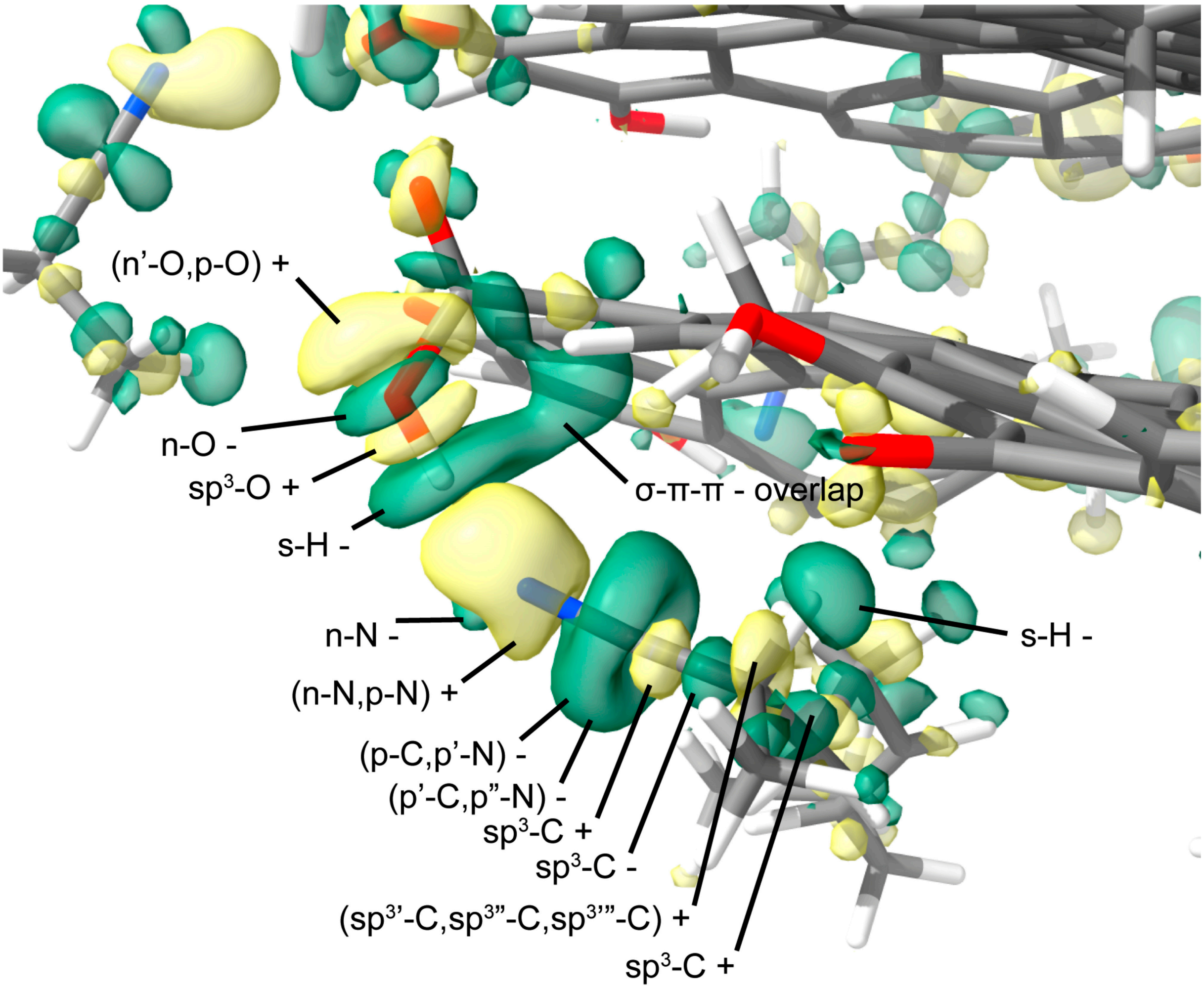

Figure S9: Changes of the electronic density because of a hydrogen bond formation between an rGO carboxyl group and a PBDAN nitrile group. Loses are symbolized with a (-) and are in green. Gains are symbolized with a (+) and are in yellow. Mixing of MOs with different geometrical symmetry occurs, because those are canonical SCF orbitals.

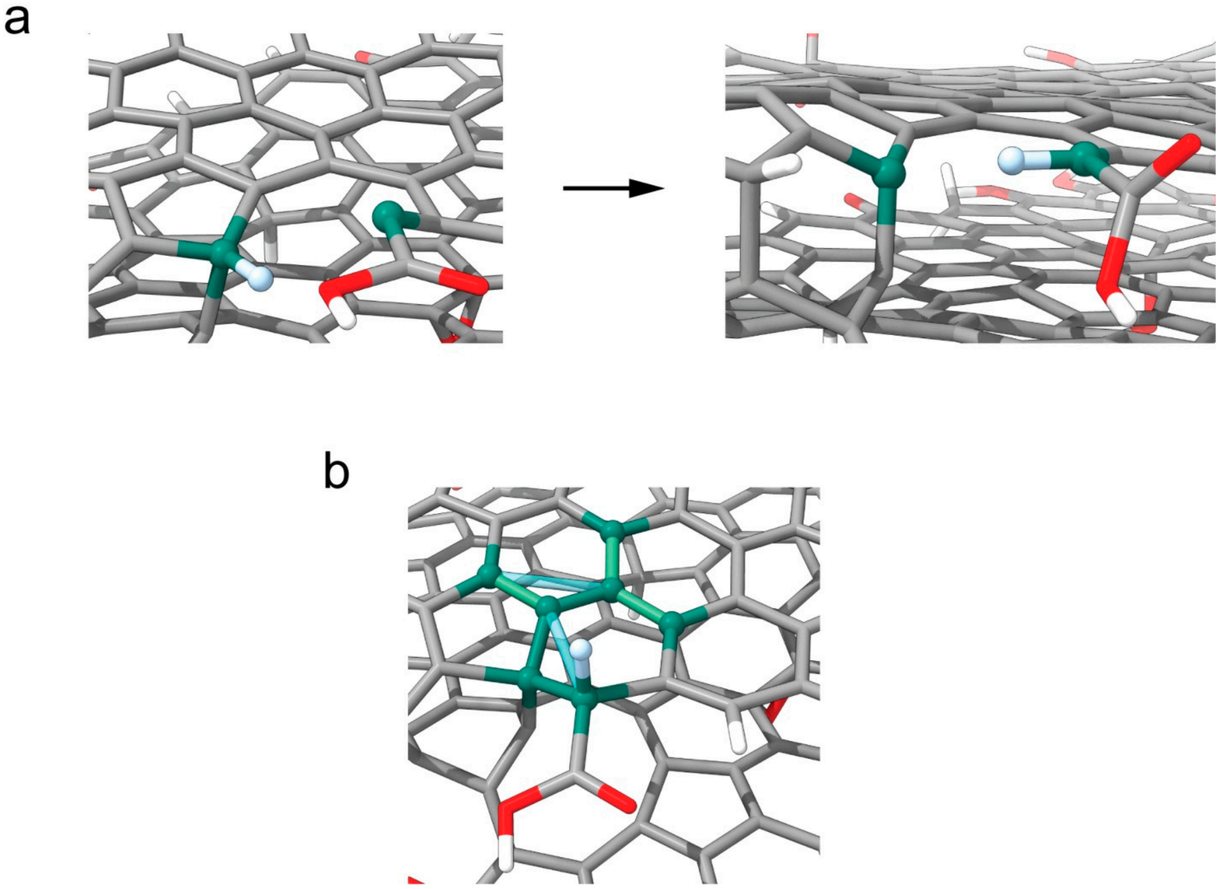

Figure S10: (a) a reaction of hydrogen transfer between two of the C atoms in the rGO layer fragment, which undergoes self-repair (section *3.4* in the main text). (b) formed and cleaved bonds during the self-repair. Green designates unaltered bonds. Transparent light blue designates cleaved bonds. Light-green designates formed bonds.
